# Supplementary material for: Modeling glioblastoma heterogeneity as a dynamic network of cell states
Source: Mol Syst Biol. 2021 Sep 16;17(9):e10105. doi: 10.15252/msb.202010105 (PMC8444284; doi:10.15252/msb.202010105)
Supplement: Supplementary file 6 — Source Data for Figure 5 [file MSB-17-e10105-s004.zip › Figure5A_sourcedata/GSEA_3071/nosigdb_stateV.GseaPreranked.1622795159954/RICHARDS_NOWAKOWSKI_SCIENCE_2017_MURAL_UPREG.html]

Details for gene set RICHARDS\_NOWAKOWSKI\_SCIENCE\_2017\_MURAL\_UPREG[GSEA]

|  || Dataset | state43071 |
| Phenotype | NoPhenotypeAvailable |
| Upregulated in class | na\_neg |
| GeneSet | RICHARDS\_NOWAKOWSKI\_SCIENCE\_2017\_MURAL\_UPREG |
| Enrichment Score (ES) | -0.46815586 |
| Normalized Enrichment Score (NES) | -3.3023627 |
| Nominal p-value | 0.0 |
| FDR q-value | 0.0 |
| FWER p-Value | 0.0 |
Table: GSEA Results Summary

  

Fig 1: Enrichment plot: RICHARDS\_NOWAKOWSKI\_SCIENCE\_2017\_MURAL\_UPREG      
 Profile of the Running ES Score & Positions of GeneSet Members on the Rank Ordered List

  

| PROBE | GENE SYMBOL | GENE\_TITLE | RANK IN GENE LIST | RANK METRIC SCORE | RUNNING ES | CORE ENRICHMENT || 1 | EMP2 |  |  | 59 | 1.511 | -0.0071 | No |
| 2 | PTN |  |  | 70 | 1.482 | -0.0043 | No |
| 3 | KLHL23 |  |  | 83 | 1.421 | -0.0021 | No |
| 4 | SLC1A3 |  |  | 110 | 1.341 | -0.0030 | No |
| 5 | GPM6B |  |  | 121 | 1.326 | -0.0007 | No |
| 6 | CADM4 |  |  | 144 | 1.284 | -0.0010 | No |
| 7 | TSPAN12 |  |  | 149 | 1.276 | 0.0023 | No |
| 8 | SPECC1 |  |  | 216 | 1.153 | -0.0074 | No |
| 9 | LIMA1 |  |  | 264 | 1.098 | -0.0135 | No |
| 10 | DAG1 |  |  | 267 | 1.095 | -0.0103 | No |
| 11 | MAP3K1 |  |  | 288 | 1.077 | -0.0108 | No |
| 12 | INPP4B |  |  | 319 | 1.047 | -0.0136 | No |
| 13 | NT5DC2 |  |  | 333 | 1.036 | -0.0128 | No |
| 14 | RGS16 |  |  | 348 | 1.018 | -0.0123 | No |
| 15 | MCAM |  |  | 370 | 0.980 | -0.0134 | No |
| 16 | APOLD1 |  |  | 372 | 0.980 | -0.0104 | No |
| 17 | SPRY1 |  |  | 389 | 0.959 | -0.0106 | No |
| 18 | AMOTL1 |  |  | 426 | 0.927 | -0.0149 | No |
| 19 | COBLL1 |  |  | 432 | 0.921 | -0.0129 | No |
| 20 | AKAP12 |  |  | 487 | 0.878 | -0.0211 | No |
| 21 | A2M |  |  | 532 | 0.846 | -0.0273 | No |
| 22 | CCDC102B |  |  | 551 | 0.830 | -0.0283 | No |
| 23 | APBB2 |  |  | 617 | 0.790 | -0.0391 | No |
| 24 | PLAT |  |  | 651 | 0.773 | -0.0433 | No |
| 25 | C18orf54 |  |  | 669 | 0.765 | -0.0443 | No |
| 26 | MBNL3 |  |  | 692 | 0.750 | -0.0463 | No |
| 27 | PTPRG |  |  | 693 | 0.750 | -0.0439 | No |
| 28 | NINJ1 |  |  | 695 | 0.748 | -0.0416 | No |
| 29 | TANC1 |  |  | 697 | 0.747 | -0.0394 | No |
| 30 | JAM3 |  |  | 704 | 0.745 | -0.0381 | No |
| 31 | PMEPA1 |  |  | 718 | 0.740 | -0.0384 | No |
| 32 | ADAMTS9 |  |  | 726 | 0.733 | -0.0374 | No |
| 33 | CD9 |  |  | 738 | 0.728 | -0.0373 | No |
| 34 | FAT1 |  |  | 740 | 0.727 | -0.0351 | No |
| 35 | JUND |  |  | 802 | 0.700 | -0.0453 | No |
| 36 | TRIB2 |  |  | 827 | 0.688 | -0.0480 | No |
| 37 | ISYNA1 |  |  | 848 | 0.679 | -0.0498 | No |
| 38 | PLCE1 |  |  | 854 | 0.676 | -0.0486 | No |
| 39 | RBPMS |  |  | 855 | 0.676 | -0.0464 | No |
| 40 | C11orf96 |  |  | 876 | 0.668 | -0.0483 | No |
| 41 | RBM6 |  |  | 914 | 0.653 | -0.0538 | No |
| 42 | FARP1 |  |  | 956 | 0.638 | -0.0601 | No |
| 43 | NBL1 |  |  | 973 | 0.630 | -0.0613 | No |
| 44 | SEMA5A |  |  | 974 | 0.630 | -0.0592 | No |
| 45 | RBP1 |  |  | 984 | 0.624 | -0.0590 | No |
| 46 | CACNB2 |  |  | 985 | 0.624 | -0.0570 | No |
| 47 | STT3B |  |  | 1007 | 0.619 | -0.0593 | No |
| 48 | COL1A2 |  |  | 1028 | 0.614 | -0.0614 | No |
| 49 | BCHE |  |  | 1116 | 0.588 | -0.0773 | No |
| 50 | TNFRSF21 |  |  | 1166 | 0.573 | -0.0855 | No |
| 51 | GNG11 |  |  | 1219 | 0.556 | -0.0943 | No |
| 52 | ITM2B |  |  | 1244 | 0.547 | -0.0974 | No |
| 53 | ASAP2 |  |  | 1271 | 0.539 | -0.1010 | No |
| 54 | KCNE4 |  |  | 1272 | 0.539 | -0.0992 | No |
| 55 | ITPR1 |  |  | 1282 | 0.537 | -0.0993 | No |
| 56 | SPRY4 |  |  | 1283 | 0.536 | -0.0975 | No |
| 57 | EFEMP2 |  |  | 1300 | 0.532 | -0.0991 | No |
| 58 | FCGRT |  |  | 1328 | 0.526 | -0.1029 | No |
| 59 | NDUFAF3 |  |  | 1363 | 0.516 | -0.1082 | No |
| 60 | TIMP2 |  |  | 1422 | 0.498 | -0.1185 | No |
| 61 | LMO2 |  |  | 1427 | 0.498 | -0.1176 | No |
| 62 | PLXDC1 |  |  | 1487 | 0.483 | -0.1282 | No |
| 63 | ITPR2 |  |  | 1543 | 0.471 | -0.1379 | No |
| 64 | PLEKHH2 |  |  | 1595 | 0.461 | -0.1469 | No |
| 65 | ZEB1 |  |  | 1605 | 0.459 | -0.1472 | No |
| 66 | TSPAN7 |  |  | 1669 | 0.442 | -0.1587 | No |
| 67 | PHF2 |  |  | 1685 | 0.439 | -0.1603 | No |
| 68 | CRTAP |  |  | 1686 | 0.439 | -0.1589 | No |
| 69 | EGR1 |  |  | 1691 | 0.438 | -0.1583 | No |
| 70 | PAG1 |  |  | 1703 | 0.435 | -0.1591 | No |
| 71 | CALCRL |  |  | 1737 | 0.429 | -0.1644 | No |
| 72 | AP2M1 |  |  | 1744 | 0.428 | -0.1643 | No |
| 73 | SEPT4 |  |  | 1753 | 0.427 | -0.1645 | No |
| 74 | GOLIM4 |  |  | 1754 | 0.427 | -0.1631 | No |
| 75 | SPCS1 |  |  | 1778 | 0.422 | -0.1664 | No |
| 76 | WWTR1 |  |  | 1794 | 0.419 | -0.1681 | No |
| 77 | CDK14 |  |  | 1822 | 0.415 | -0.1723 | No |
| 78 | CNP |  |  | 1879 | 0.406 | -0.1825 | No |
| 79 | FRZB |  |  | 1884 | 0.406 | -0.1820 | No |
| 80 | SNRK |  |  | 1939 | 0.398 | -0.1918 | No |
| 81 | TLE1 |  |  | 1947 | 0.397 | -0.1919 | No |
| 82 | LAMB2 |  |  | 2003 | 0.387 | -0.2019 | No |
| 83 | MANF |  |  | 2023 | 0.384 | -0.2045 | No |
| 84 | DENND5A |  |  | 2038 | 0.382 | -0.2062 | No |
| 85 | CDH6 |  |  | 2101 | 0.374 | -0.2177 | No |
| 86 | ZIC1 |  |  | 2150 | 0.368 | -0.2263 | No |
| 87 | ANXA1 |  |  | 2165 | 0.366 | -0.2280 | No |
| 88 | LDHB |  |  | 2219 | 0.360 | -0.2377 | No |
| 89 | CHRAC1 |  |  | 2229 | 0.359 | -0.2383 | No |
| 90 | ASNA1 |  |  | 2264 | 0.355 | -0.2442 | No |
| 91 | NID1 |  |  | 2297 | 0.350 | -0.2496 | No |
| 92 | EEF1D |  |  | 2307 | 0.349 | -0.2503 | No |
| 93 | MRC2 |  |  | 2378 | 0.338 | -0.2635 | No |
| 94 | ROCK2 |  |  | 2385 | 0.337 | -0.2637 | No |
| 95 | CRIP2 |  |  | 2419 | 0.332 | -0.2693 | No |
| 96 | TOB1 |  |  | 2442 | 0.330 | -0.2728 | No |
| 97 | RHOA |  |  | 2519 | 0.321 | -0.2873 | No |
| 98 | CAMK2D |  |  | 2520 | 0.321 | -0.2863 | No |
| 99 | FOS |  |  | 2529 | 0.319 | -0.2869 | No |
| 100 | ITM2C |  |  | 2558 | 0.316 | -0.2916 | No |
| 101 | ECM1 |  |  | 2564 | 0.315 | -0.2916 | No |
| 102 | HSPA12A |  |  | 2582 | 0.313 | -0.2940 | No |
| 103 | COX6C |  |  | 2583 | 0.313 | -0.2930 | No |
| 104 | PDE8B |  |  | 2594 | 0.312 | -0.2940 | No |
| 105 | FNDC3B |  |  | 2617 | 0.308 | -0.2975 | No |
| 106 | COLGALT1 |  |  | 2623 | 0.307 | -0.2975 | No |
| 107 | SYPL1 |  |  | 2625 | 0.307 | -0.2967 | No |
| 108 | SASH1 |  |  | 2634 | 0.306 | -0.2974 | No |
| 109 | ADAMTS4 |  |  | 2658 | 0.304 | -0.3011 | No |
| 110 | PRRX1 |  |  | 2674 | 0.302 | -0.3032 | No |
| 111 | AGFG1 |  |  | 2695 | 0.300 | -0.3063 | No |
| 112 | SRSF3 |  |  | 2732 | 0.297 | -0.3127 | No |
| 113 | COL9A1 |  |  | 2756 | 0.294 | -0.3165 | No |
| 114 | MTUS1 |  |  | 2764 | 0.293 | -0.3170 | No |
| 115 | TIPARP |  |  | 2768 | 0.292 | -0.3166 | No |
| 116 | SLC25A5 |  |  | 2796 | 0.290 | -0.3212 | No |
| 117 | SH3PXD2A |  |  | 2831 | 0.286 | -0.3272 | No |
| 118 | PRDM1 |  |  | 2921 | 0.277 | -0.3446 | No |
| 119 | CERKL |  |  | 2927 | 0.276 | -0.3447 | No |
| 120 | ELK4 |  |  | 2971 | 0.272 | -0.3527 | No |
| 121 | CACNB4 |  |  | 2988 | 0.271 | -0.3550 | No |
| 122 | COX8A |  |  | 2998 | 0.270 | -0.3560 | No |
| 123 | CALU |  |  | 3001 | 0.269 | -0.3555 | No |
| 124 | PPM1B |  |  | 3007 | 0.268 | -0.3557 | No |
| 125 | GUCY1A2 |  |  | 3017 | 0.267 | -0.3567 | No |
| 126 | SEPT11 |  |  | 3027 | 0.266 | -0.3576 | No |
| 127 | STARD9 |  |  | 3044 | 0.264 | -0.3600 | No |
| 128 | PROS1 |  |  | 3097 | 0.260 | -0.3699 | No |
| 129 | CHST2 |  |  | 3126 | 0.258 | -0.3748 | No |
| 130 | DNAJB1 |  |  | 3143 | 0.256 | -0.3772 | No |
| 131 | HSPA2 |  |  | 3149 | 0.255 | -0.3774 | No |
| 132 | ATP1B3 |  |  | 3158 | 0.254 | -0.3782 | No |
| 133 | RORA |  |  | 3171 | 0.253 | -0.3798 | No |
| 134 | TECR |  |  | 3183 | 0.252 | -0.3813 | No |
| 135 | PRICKLE2 |  |  | 3184 | 0.251 | -0.3804 | No |
| 136 | DISC1 |  |  | 3188 | 0.251 | -0.3802 | No |
| 137 | CTTN |  |  | 3201 | -0.251 | -0.3819 | No |
| 138 | PSME1 |  |  | 3202 | -0.251 | -0.3810 | No |
| 139 | PID1 |  |  | 3215 | -0.253 | -0.3827 | No |
| 140 | MYO1B |  |  | 3217 | -0.254 | -0.3820 | No |
| 141 | STOM |  |  | 3230 | -0.256 | -0.3837 | No |
| 142 | S1PR2 |  |  | 3231 | -0.257 | -0.3828 | No |
| 143 | IL6ST |  |  | 3233 | -0.257 | -0.3822 | No |
| 144 | COL18A1 |  |  | 3251 | -0.259 | -0.3848 | No |
| 145 | SLK |  |  | 3264 | -0.261 | -0.3864 | No |
| 146 | TMEM109 |  |  | 3265 | -0.261 | -0.3856 | No |
| 147 | PARP8 |  |  | 3279 | -0.262 | -0.3874 | No |
| 148 | KITLG |  |  | 3280 | -0.262 | -0.3865 | No |
| 149 | ATP6V1G1 |  |  | 3316 | -0.270 | -0.3928 | No |
| 150 | TRIM5 |  |  | 3328 | -0.271 | -0.3942 | No |
| 151 | FKBP10 |  |  | 3330 | -0.272 | -0.3935 | No |
| 152 | RNF152 |  |  | 3340 | -0.274 | -0.3944 | No |
| 153 | ECE1 |  |  | 3354 | -0.275 | -0.3962 | No |
| 154 | UBE2Z |  |  | 3393 | -0.280 | -0.4031 | No |
| 155 | TSC22D3 |  |  | 3396 | -0.281 | -0.4026 | No |
| 156 | GCNT2 |  |  | 3439 | -0.288 | -0.4102 | No |
| 157 | WDR1 |  |  | 3447 | -0.289 | -0.4107 | No |
| 158 | LAMA2 |  |  | 3449 | -0.289 | -0.4100 | No |
| 159 | PFDN5 |  |  | 3451 | -0.290 | -0.4092 | No |
| 160 | NUMB |  |  | 3472 | -0.295 | -0.4124 | No |
| 161 | HSPA1A |  |  | 3494 | -0.299 | -0.4157 | No |
| 162 | ZBTB38 |  |  | 3495 | -0.299 | -0.4147 | No |
| 163 | PPIB |  |  | 3507 | -0.301 | -0.4160 | No |
| 164 | TBX15 |  |  | 3547 | -0.307 | -0.4230 | No |
| 165 | AGTRAP |  |  | 3553 | -0.308 | -0.4230 | No |
| 166 | KLF10 |  |  | 3555 | -0.308 | -0.4222 | No |
| 167 | KTN1 |  |  | 3560 | -0.309 | -0.4220 | No |
| 168 | GSTK1 |  |  | 3570 | -0.311 | -0.4228 | No |
| 169 | IFITM2 |  |  | 3585 | -0.312 | -0.4247 | No |
| 170 | WDR41 |  |  | 3589 | -0.314 | -0.4243 | No |
| 171 | TPM4 |  |  | 3591 | -0.314 | -0.4234 | No |
| 172 | HADHB |  |  | 3598 | -0.315 | -0.4236 | No |
| 173 | ITGB1 |  |  | 3614 | -0.317 | -0.4257 | No |
| 174 | STAM2 |  |  | 3618 | -0.318 | -0.4252 | No |
| 175 | BHLHE40 |  |  | 3620 | -0.318 | -0.4244 | No |
| 176 | KANK2 |  |  | 3634 | -0.321 | -0.4260 | No |
| 177 | MTO1 |  |  | 3652 | -0.323 | -0.4284 | No |
| 178 | CALCOCO2 |  |  | 3677 | -0.327 | -0.4323 | No |
| 179 | GMDS |  |  | 3681 | -0.328 | -0.4318 | No |
| 180 | MRPL24 |  |  | 3690 | -0.330 | -0.4324 | No |
| 181 | GSN |  |  | 3695 | -0.331 | -0.4321 | No |
| 182 | TAPBP |  |  | 3712 | -0.333 | -0.4343 | No |
| 183 | EVI5 |  |  | 3716 | -0.334 | -0.4338 | No |
| 184 | CPT1A |  |  | 3730 | -0.336 | -0.4354 | No |
| 185 | CNN3 |  |  | 3751 | -0.339 | -0.4384 | No |
| 186 | TNS1 |  |  | 3761 | -0.342 | -0.4391 | No |
| 187 | SVIL |  |  | 3763 | -0.342 | -0.4382 | No |
| 188 | CYSTM1 |  |  | 3782 | -0.346 | -0.4407 | No |
| 189 | SERINC5 |  |  | 3807 | -0.350 | -0.4445 | No |
| 190 | NDUFA1 |  |  | 3818 | -0.353 | -0.4454 | No |
| 191 | TMOD3 |  |  | 3820 | -0.353 | -0.4445 | No |
| 192 | SDHD |  |  | 3822 | -0.354 | -0.4435 | No |
| 193 | SSR3 |  |  | 3832 | -0.355 | -0.4442 | No |
| 194 | RPL10A |  |  | 3846 | -0.358 | -0.4457 | No |
| 195 | MAFF |  |  | 3849 | -0.359 | -0.4449 | No |
| 196 | CAPZA1 |  |  | 3859 | -0.360 | -0.4456 | No |
| 197 | YBX3 |  |  | 3863 | -0.361 | -0.4450 | No |
| 198 | KLF4 |  |  | 3866 | -0.361 | -0.4442 | No |
| 199 | IFIT3 |  |  | 3874 | -0.362 | -0.4445 | No |
| 200 | VSTM4 |  |  | 3879 | -0.363 | -0.4441 | No |
| 201 | C4orf3 |  |  | 3884 | -0.364 | -0.4437 | No |
| 202 | APOBEC3C |  |  | 3885 | -0.364 | -0.4425 | No |
| 203 | DR1 |  |  | 3888 | -0.365 | -0.4417 | No |
| 204 | FOXC1 |  |  | 3889 | -0.365 | -0.4405 | No |
| 205 | PABPC1 |  |  | 3893 | -0.366 | -0.4399 | No |
| 206 | EPB41L2 |  |  | 3911 | -0.369 | -0.4422 | No |
| 207 | NBPF14 |  |  | 3939 | -0.376 | -0.4465 | No |
| 208 | PKIG |  |  | 3947 | -0.377 | -0.4467 | No |
| 209 | COL5A3 |  |  | 3975 | -0.383 | -0.4510 | No |
| 210 | JAK1 |  |  | 4019 | -0.393 | -0.4585 | No |
| 211 | CD59 |  |  | 4024 | -0.394 | -0.4581 | No |
| 212 | JUNB |  |  | 4027 | -0.394 | -0.4572 | No |
| 213 | EPAS1 |  |  | 4032 | -0.395 | -0.4567 | No |
| 214 | YAP1 |  |  | 4041 | -0.398 | -0.4570 | No |
| 215 | EHD2 |  |  | 4051 | -0.399 | -0.4576 | No |
| 216 | FOXF2 |  |  | 4055 | -0.400 | -0.4569 | No |
| 217 | ETF1 |  |  | 4072 | -0.403 | -0.4588 | No |
| 218 | PSMB6 |  |  | 4090 | -0.407 | -0.4610 | No |
| 219 | NDUFB3 |  |  | 4102 | -0.410 | -0.4619 | No |
| 220 | RPL13A |  |  | 4111 | -0.412 | -0.4622 | No |
| 221 | LAMB1 |  |  | 4115 | -0.413 | -0.4614 | No |
| 222 | FKBP1A |  |  | 4124 | -0.415 | -0.4617 | No |
| 223 | HIF1A |  |  | 4130 | -0.417 | -0.4614 | No |
| 224 | JUN |  |  | 4135 | -0.418 | -0.4608 | No |
| 225 | LDHA |  |  | 4145 | -0.421 | -0.4613 | No |
| 226 | SPATS2L |  |  | 4148 | -0.422 | -0.4603 | No |
| 227 | RPLP2 |  |  | 4156 | -0.426 | -0.4603 | No |
| 228 | ATP8B1 |  |  | 4161 | -0.427 | -0.4598 | No |
| 229 | SERINC3 |  |  | 4172 | -0.429 | -0.4604 | No |
| 230 | PXDN |  |  | 4176 | -0.430 | -0.4596 | No |
| 231 | TGFB1I1 |  |  | 4177 | -0.430 | -0.4582 | No |
| 232 | PLAC9 |  |  | 4197 | -0.437 | -0.4607 | No |
| 233 | PHACTR2 |  |  | 4213 | -0.444 | -0.4623 | No |
| 234 | MYADM |  |  | 4217 | -0.445 | -0.4614 | No |
| 235 | COMT |  |  | 4243 | -0.453 | -0.4651 | No |
| 236 | ARPC2 |  |  | 4259 | -0.458 | -0.4666 | Yes |
| 237 | FSTL1 |  |  | 4262 | -0.460 | -0.4655 | Yes |
| 238 | TRIM38 |  |  | 4264 | -0.461 | -0.4642 | Yes |
| 239 | MYL12B |  |  | 4267 | -0.462 | -0.4631 | Yes |
| 240 | PELO |  |  | 4268 | -0.463 | -0.4616 | Yes |
| 241 | PDE5A |  |  | 4271 | -0.465 | -0.4605 | Yes |
| 242 | ATL3 |  |  | 4273 | -0.466 | -0.4592 | Yes |
| 243 | HTRA3 |  |  | 4274 | -0.466 | -0.4576 | Yes |
| 244 | ASPN |  |  | 4291 | -0.471 | -0.4594 | Yes |
| 245 | RPL23A |  |  | 4295 | -0.471 | -0.4584 | Yes |
| 246 | C14orf119 |  |  | 4300 | -0.472 | -0.4577 | Yes |
| 247 | ATP6V0E1 |  |  | 4302 | -0.473 | -0.4564 | Yes |
| 248 | ELOVL1 |  |  | 4305 | -0.474 | -0.4552 | Yes |
| 249 | ARHGAP24 |  |  | 4315 | -0.476 | -0.4555 | Yes |
| 250 | F2R |  |  | 4316 | -0.476 | -0.4539 | Yes |
| 251 | PSMB8 |  |  | 4318 | -0.477 | -0.4526 | Yes |
| 252 | UNC5B |  |  | 4324 | -0.478 | -0.4520 | Yes |
| 253 | APOL2 |  |  | 4331 | -0.481 | -0.4517 | Yes |
| 254 | CD99 |  |  | 4339 | -0.484 | -0.4515 | Yes |
| 255 | B2M |  |  | 4341 | -0.485 | -0.4501 | Yes |
| 256 | MEF2C |  |  | 4351 | -0.487 | -0.4504 | Yes |
| 257 | PARVA |  |  | 4352 | -0.487 | -0.4488 | Yes |
| 258 | EDNRA |  |  | 4361 | -0.489 | -0.4488 | Yes |
| 259 | HNMT |  |  | 4379 | -0.494 | -0.4507 | Yes |
| 260 | POLR2L |  |  | 4382 | -0.496 | -0.4495 | Yes |
| 261 | SYNE1 |  |  | 4397 | -0.500 | -0.4507 | Yes |
| 262 | MYO1C |  |  | 4421 | -0.508 | -0.4537 | Yes |
| 263 | PSME2 |  |  | 4422 | -0.508 | -0.4521 | Yes |
| 264 | IFITM3 |  |  | 4424 | -0.509 | -0.4506 | Yes |
| 265 | FERMT2 |  |  | 4445 | -0.519 | -0.4530 | Yes |
| 266 | OST4 |  |  | 4463 | -0.524 | -0.4548 | Yes |
| 267 | PDZD2 |  |  | 4466 | -0.525 | -0.4535 | Yes |
| 268 | ARL1 |  |  | 4471 | -0.525 | -0.4526 | Yes |
| 269 | RPS5 |  |  | 4475 | -0.529 | -0.4514 | Yes |
| 270 | TJP1 |  |  | 4489 | -0.536 | -0.4523 | Yes |
| 271 | DCTD |  |  | 4495 | -0.539 | -0.4516 | Yes |
| 272 | RALB |  |  | 4496 | -0.539 | -0.4498 | Yes |
| 273 | CCDC80 |  |  | 4510 | -0.546 | -0.4507 | Yes |
| 274 | ADORA2B |  |  | 4535 | -0.556 | -0.4538 | Yes |
| 275 | SLC38A2 |  |  | 4536 | -0.557 | -0.4520 | Yes |
| 276 | SSR4 |  |  | 4540 | -0.558 | -0.4507 | Yes |
| 277 | SP140L |  |  | 4545 | -0.561 | -0.4497 | Yes |
| 278 | CYTH3 |  |  | 4547 | -0.563 | -0.4481 | Yes |
| 279 | BMP1 |  |  | 4559 | -0.569 | -0.4485 | Yes |
| 280 | RPS7 |  |  | 4574 | -0.574 | -0.4494 | Yes |
| 281 | NOP10 |  |  | 4575 | -0.574 | -0.4476 | Yes |
| 282 | TPT1 |  |  | 4579 | -0.576 | -0.4463 | Yes |
| 283 | ISG15 |  |  | 4586 | -0.579 | -0.4456 | Yes |
| 284 | PIP4K2A |  |  | 4591 | -0.581 | -0.4445 | Yes |
| 285 | ALX4 |  |  | 4613 | -0.599 | -0.4469 | Yes |
| 286 | ANXA5 |  |  | 4616 | -0.601 | -0.4453 | Yes |
| 287 | TXNIP |  |  | 4618 | -0.604 | -0.4435 | Yes |
| 288 | UACA |  |  | 4621 | -0.604 | -0.4419 | Yes |
| 289 | IGFBP7 |  |  | 4622 | -0.604 | -0.4400 | Yes |
| 290 | S1PR3 |  |  | 4625 | -0.605 | -0.4384 | Yes |
| 291 | TWISTNB |  |  | 4630 | -0.609 | -0.4372 | Yes |
| 292 | NR3C1 |  |  | 4673 | -0.630 | -0.4438 | Yes |
| 293 | PLTP |  |  | 4682 | -0.634 | -0.4433 | Yes |
| 294 | P4HA1 |  |  | 4689 | -0.641 | -0.4424 | Yes |
| 295 | NTM |  |  | 4695 | -0.643 | -0.4414 | Yes |
| 296 | MSN |  |  | 4698 | -0.644 | -0.4396 | Yes |
| 297 | KDELR2 |  |  | 4700 | -0.645 | -0.4377 | Yes |
| 298 | ITGA1 |  |  | 4706 | -0.647 | -0.4366 | Yes |
| 299 | HSPA5 |  |  | 4714 | -0.652 | -0.4359 | Yes |
| 300 | RPS14 |  |  | 4715 | -0.653 | -0.4338 | Yes |
| 301 | TLN1 |  |  | 4718 | -0.658 | -0.4320 | Yes |
| 302 | RPL23 |  |  | 4726 | -0.662 | -0.4313 | Yes |
| 303 | RSU1 |  |  | 4738 | -0.670 | -0.4313 | Yes |
| 304 | SLC29A1 |  |  | 4740 | -0.671 | -0.4293 | Yes |
| 305 | RPS6 |  |  | 4751 | -0.678 | -0.4292 | Yes |
| 306 | PLS3 |  |  | 4771 | -0.692 | -0.4308 | Yes |
| 307 | DOCK10 |  |  | 4777 | -0.696 | -0.4295 | Yes |
| 308 | LIMS1 |  |  | 4781 | -0.698 | -0.4279 | Yes |
| 309 | DYRK2 |  |  | 4782 | -0.699 | -0.4256 | Yes |
| 310 | LGALS1 |  |  | 4784 | -0.699 | -0.4235 | Yes |
| 311 | CTSL |  |  | 4785 | -0.701 | -0.4212 | Yes |
| 312 | RPL13 |  |  | 4787 | -0.702 | -0.4191 | Yes |
| 313 | DUSP5 |  |  | 4796 | -0.707 | -0.4184 | Yes |
| 314 | MT2A |  |  | 4799 | -0.714 | -0.4164 | Yes |
| 315 | PCDH18 |  |  | 4803 | -0.717 | -0.4147 | Yes |
| 316 | NEK7 |  |  | 4807 | -0.719 | -0.4129 | Yes |
| 317 | POMP |  |  | 4812 | -0.725 | -0.4114 | Yes |
| 318 | ARPC1B |  |  | 4818 | -0.730 | -0.4100 | Yes |
| 319 | RHOB |  |  | 4820 | -0.731 | -0.4078 | Yes |
| 320 | HBEGF |  |  | 4822 | -0.734 | -0.4056 | Yes |
| 321 | DYNLT3 |  |  | 4823 | -0.734 | -0.4032 | Yes |
| 322 | EBF1 |  |  | 4831 | -0.738 | -0.4022 | Yes |
| 323 | DNAJB4 |  |  | 4833 | -0.739 | -0.4000 | Yes |
| 324 | PLOD1 |  |  | 4854 | -0.757 | -0.4016 | Yes |
| 325 | DAD1 |  |  | 4860 | -0.763 | -0.4001 | Yes |
| 326 | SLC3A2 |  |  | 4861 | -0.765 | -0.3976 | Yes |
| 327 | RHOC |  |  | 4868 | -0.776 | -0.3963 | Yes |
| 328 | PXDC1 |  |  | 4872 | -0.780 | -0.3943 | Yes |
| 329 | THY1 |  |  | 4874 | -0.781 | -0.3920 | Yes |
| 330 | TLCD2 |  |  | 4880 | -0.786 | -0.3904 | Yes |
| 331 | MAGED2 |  |  | 4884 | -0.793 | -0.3884 | Yes |
| 332 | RPS12 |  |  | 4888 | -0.795 | -0.3864 | Yes |
| 333 | GPRC5C |  |  | 4890 | -0.801 | -0.3840 | Yes |
| 334 | ARHGAP26 |  |  | 4894 | -0.804 | -0.3820 | Yes |
| 335 | CLIC1 |  |  | 4899 | -0.809 | -0.3801 | Yes |
| 336 | NR2F2 |  |  | 4917 | -0.830 | -0.3809 | Yes |
| 337 | ZFP36L1 |  |  | 4929 | -0.843 | -0.3804 | Yes |
| 338 | KLF6 |  |  | 4933 | -0.849 | -0.3782 | Yes |
| 339 | ITIH5 |  |  | 4934 | -0.850 | -0.3754 | Yes |
| 340 | NOTCH3 |  |  | 4936 | -0.852 | -0.3728 | Yes |
| 341 | SLC2A3 |  |  | 4942 | -0.859 | -0.3710 | Yes |
| 342 | ITPRIPL2 |  |  | 4943 | -0.860 | -0.3682 | Yes |
| 343 | TMEM263 |  |  | 4946 | -0.861 | -0.3658 | Yes |
| 344 | ZFP36L2 |  |  | 4950 | -0.867 | -0.3635 | Yes |
| 345 | CALD1 |  |  | 4960 | -0.877 | -0.3625 | Yes |
| 346 | PPP1R15A |  |  | 4966 | -0.882 | -0.3606 | Yes |
| 347 | MIR22HG |  |  | 4968 | -0.885 | -0.3579 | Yes |
| 348 | DDR2 |  |  | 4971 | -0.886 | -0.3554 | Yes |
| 349 | TNS3 |  |  | 4973 | -0.890 | -0.3527 | Yes |
| 350 | RRBP1 |  |  | 4981 | -0.898 | -0.3512 | Yes |
| 351 | PAPSS2 |  |  | 4983 | -0.901 | -0.3484 | Yes |
| 352 | SBDS |  |  | 4986 | -0.908 | -0.3459 | Yes |
| 353 | HSPB1 |  |  | 4992 | -0.920 | -0.3439 | Yes |
| 354 | SYTL2 |  |  | 5001 | -0.932 | -0.3424 | Yes |
| 355 | CAP1 |  |  | 5002 | -0.934 | -0.3394 | Yes |
| 356 | ADA |  |  | 5005 | -0.945 | -0.3367 | Yes |
| 357 | CTSB |  |  | 5007 | -0.948 | -0.3338 | Yes |
| 358 | TNFAIP8 |  |  | 5009 | -0.949 | -0.3308 | Yes |
| 359 | ITGA10 |  |  | 5016 | -0.960 | -0.3289 | Yes |
| 360 | OSTC |  |  | 5019 | -0.972 | -0.3261 | Yes |
| 361 | PRKG1 |  |  | 5028 | -0.988 | -0.3245 | Yes |
| 362 | PAWR |  |  | 5033 | -1.000 | -0.3221 | Yes |
| 363 | DLC1 |  |  | 5036 | -1.013 | -0.3191 | Yes |
| 364 | ANXA2 |  |  | 5055 | -1.036 | -0.3194 | Yes |
| 365 | SERPINH1 |  |  | 5059 | -1.050 | -0.3166 | Yes |
| 366 | TWIST1 |  |  | 5060 | -1.052 | -0.3131 | Yes |
| 367 | RASL12 |  |  | 5074 | -1.084 | -0.3123 | Yes |
| 368 | CEBPB |  |  | 5076 | -1.088 | -0.3089 | Yes |
| 369 | CPED1 |  |  | 5078 | -1.090 | -0.3055 | Yes |
| 370 | KLF2 |  |  | 5083 | -1.110 | -0.3027 | Yes |
| 371 | MYH9 |  |  | 5090 | -1.125 | -0.3002 | Yes |
| 372 | COL5A2 |  |  | 5112 | -1.189 | -0.3006 | Yes |
| 373 | PDLIM1 |  |  | 5114 | -1.191 | -0.2969 | Yes |
| 374 | NUPR1 |  |  | 5123 | -1.217 | -0.2945 | Yes |
| 375 | SP100 |  |  | 5126 | -1.230 | -0.2909 | Yes |
| 376 | FAM114A1 |  |  | 5127 | -1.230 | -0.2869 | Yes |
| 377 | RBMS1 |  |  | 5129 | -1.240 | -0.2830 | Yes |
| 378 | AXL |  |  | 5132 | -1.249 | -0.2793 | Yes |
| 379 | TIMP1 |  |  | 5134 | -1.251 | -0.2754 | Yes |
| 380 | OLFML2A |  |  | 5140 | -1.270 | -0.2722 | Yes |
| 381 | LMNA |  |  | 5141 | -1.277 | -0.2680 | Yes |
| 382 | CAST |  |  | 5142 | -1.277 | -0.2638 | Yes |
| 383 | MMP14 |  |  | 5146 | -1.297 | -0.2602 | Yes |
| 384 | LBH |  |  | 5153 | -1.323 | -0.2571 | Yes |
| 385 | SERPINE1 |  |  | 5161 | -1.377 | -0.2540 | Yes |
| 386 | CD248 |  |  | 5178 | -1.448 | -0.2525 | Yes |
| 387 | DDIT3 |  |  | 5180 | -1.461 | -0.2479 | Yes |
| 388 | PRSS23 |  |  | 5189 | -1.489 | -0.2447 | Yes |
| 389 | CAV2 |  |  | 5194 | -1.527 | -0.2405 | Yes |
| 390 | S100A11 |  |  | 5196 | -1.536 | -0.2356 | Yes |
| 391 | COL4A2 |  |  | 5198 | -1.545 | -0.2308 | Yes |
| 392 | TFPI |  |  | 5202 | -1.557 | -0.2262 | Yes |
| 393 | MYOF |  |  | 5207 | -1.590 | -0.2218 | Yes |
| 394 | BGN |  |  | 5216 | -1.654 | -0.2180 | Yes |
| 395 | ETS2 |  |  | 5221 | -1.671 | -0.2134 | Yes |
| 396 | VCL |  |  | 5222 | -1.678 | -0.2079 | Yes |
| 397 | OLFML3 |  |  | 5224 | -1.700 | -0.2025 | Yes |
| 398 | FRMD6 |  |  | 5228 | -1.738 | -0.1974 | Yes |
| 399 | DCN |  |  | 5233 | -1.798 | -0.1923 | Yes |
| 400 | MYL12A |  |  | 5239 | -1.826 | -0.1873 | Yes |
| 401 | SFTA1P |  |  | 5240 | -1.840 | -0.1813 | Yes |
| 402 | SNAI2 |  |  | 5241 | -1.903 | -0.1750 | Yes |
| 403 | F3 |  |  | 5242 | -1.927 | -0.1687 | Yes |
| 404 | CDH11 |  |  | 5245 | -1.943 | -0.1627 | Yes |
| 405 | TPM2 |  |  | 5247 | -1.967 | -0.1564 | Yes |
| 406 | PCOLCE |  |  | 5248 | -1.999 | -0.1499 | Yes |
| 407 | MGLL |  |  | 5249 | -2.003 | -0.1433 | Yes |
| 408 | MGP |  |  | 5252 | -2.037 | -0.1370 | Yes |
| 409 | PDGFRB |  |  | 5253 | -2.045 | -0.1303 | Yes |
| 410 | MSRB3 |  |  | 5254 | -2.046 | -0.1236 | Yes |
| 411 | COLEC12 |  |  | 5256 | -2.134 | -0.1167 | Yes |
| 412 | CFH |  |  | 5257 | -2.141 | -0.1097 | Yes |
| 413 | FBN1 |  |  | 5263 | -2.234 | -0.1034 | Yes |
| 414 | COL4A1 |  |  | 5266 | -2.279 | -0.0963 | Yes |
| 415 | SLC9A3R1 |  |  | 5271 | -2.439 | -0.0891 | Yes |
| 416 | COL5A1 |  |  | 5274 | -2.455 | -0.0815 | Yes |
| 417 | LUM |  |  | 5277 | -2.507 | -0.0736 | Yes |
| 418 | ARHGAP29 |  |  | 5278 | -2.576 | -0.0652 | Yes |
| 419 | CAV1 |  |  | 5279 | -2.605 | -0.0566 | Yes |
| 420 | MYL9 |  |  | 5280 | -2.643 | -0.0479 | Yes |
| 421 | TAGLN |  |  | 5281 | -2.669 | -0.0391 | Yes |
| 422 | COL6A1 |  |  | 5283 | -2.747 | -0.0303 | Yes |
| 423 | FN1 |  |  | 5287 | -2.866 | -0.0215 | Yes |
| 424 | EDIL3 |  |  | 5290 | -3.285 | -0.0111 | Yes |
| 425 | COL3A1 |  |  | 5292 | -3.630 | 0.0006 | Yes |
Table: GSEA details [plain text format]

  

Fig 2: RICHARDS\_NOWAKOWSKI\_SCIENCE\_2017\_MURAL\_UPREG: Random ES distribution      
 Gene set null distribution of ES for **RICHARDS\_NOWAKOWSKI\_SCIENCE\_2017\_MURAL\_UPREG**
